# Similarities and Differences in Gene Expression Networks Between the Breast Cancer Cell Line Michigan Cancer Foundation-7 and Invasive Human Breast Cancer Tissues

**DOI:** 10.3389/frai.2021.674370

**Published:** 2021-05-13

**Authors:** Vy Tran, Robert Kim, Mikhail Maertens, Thomas Hartung, Alexandra Maertens

**Affiliations:** ^1^ Department of Environmental Health and Engineering, Center for Alternatives to Animal Testing, Bloomberg School of Public Health, Johns Hopkins University, Baltimore, MD, United States; ^2^ Department of Biology, Center for Alternatives to Animal Testing–Europe, University of Konstanz, Konstanz, Germany; ^3^ Department of Environmental Health and Engineering, Doerenkamp-Zbinden Professor and Chair for Evidence-Based Toxicology, Bloomberg School of Public Health, Johns Hopkins University, Baltimore, MD, United States

**Keywords:** WGCNA, TCGA, cell line relevance, network analysis, human breast cancer tissues, MCF-7

## Abstract

Failure to adequately characterize cell lines, and understand the differences between *in vitro* and *in vivo* biology, can have serious consequences on the translatability of *in vitro* scientific studies to human clinical trials. This project focuses on the Michigan Cancer Foundation-7 (MCF-7) cells, a human breast adenocarcinoma cell line that is commonly used for *in vitro* cancer research, with over 42,000 publications in PubMed. In this study, we explore the key similarities and differences in gene expression networks of MCF-7 cell lines compared to human breast cancer tissues. We used two MCF-7 data sets, one data set collected by ARCHS4 including 1032 samples and one data set from Gene Expression Omnibus GSE50705 with 88 estradiol-treated MCF-7 samples. The human breast invasive ductal carcinoma (BRCA) data set came from The Cancer Genome Atlas, including 1212 breast tissue samples. Weighted Gene Correlation Network Analysis (WGCNA) and functional annotations of the data showed that MCF-7 cells and human breast tissues have only minimal similarity in biological processes, although some fundamental functions, such as cell cycle, are conserved. Scaled connectivity—a network topology metric—also showed drastic differences in the behavior of genes between MCF-7 and BRCA data sets. Finally, we used canSAR to compute ligand-based druggability scores of genes in the data sets, and our results suggested that using MCF-7 to study breast cancer may lead to missing important gene targets. Our comparison of the networks of MCF-7 and human breast cancer highlights the nuances of using MCF-7 to study human breast cancer and can contribute to better experimental design and result interpretation of study involving this cell line.

## Introduction

Cell lines have been extensively used as models for human biology and have contributed to many insights: from the development of vaccines and toxicology screening, to the study of disease mechanisms and treatments. Despite these achievements, there have been growing concerns about the quality of cell lines (Hartung 2007), ranging from cell-line misidentification, unreproducible studies, to failed clinical trials (Schweppe et al., 2008; Gillet et al., 2013; Hartung, 2013). In 2012, Amgen researchers attempted to replicate 53 landmark cancer papers and found that 47 studies were not reproducible (Begley and Ellis, 2012); the result is in keeping with a broader estimate that most research studies are likely to be not reproducible (Ioannidis, 2005). This leads to wasteful use of financial resources and labor, with an estimation of 28 billion dollars a year spent on irreproducible research (Freedman et al., 2015). While various reasons contribute to the irreproducibility of research, including study power, technical and biological variability, cell line reproducibility has been considered as one of the major factors contributing to the failure to reproduce preclinical studies. For instance, cell line misidentification has been a long standing problem in cell culture, with controversies for HeLa cells dating back to the 1970s (Nelson-Rees et al., 1974). In addition, the usefulness of cell lines as models for human biology has been questioned. Not all cancer cell lines have the same value as models to study cancer in humans (Gillet et al., 2013).

Michigan Cancer Foundation-7 cells (MCF-7) have been used widely in labs as a model for human breast cancer for over 40 years. It is estrogen receptor (ER)-postive, progesterone receptor (PR)-positive, poorly aggresive, and non-invasive, with low metastatic capacity (Comsa et al., 2015). Since its creation in 1973, MCF-7 has resulted in the highest number of scientific papers compared to other breast cancer cell lines (Sweeney et al., 2012), with over 42,000 publications on PubMed related to this cell line. MCF-7 has played an important role in studying estrogen receptor (ER) in tumor growth, characterization of cancer drug candidates, and endocrine disruption screening (Comsa et al., 2015). Since cancer cell lines greatly contribute to our understanding of cancer molecular mechanisms, investigating their relevance of cancer cell lines to human cancer is critical. Noticeably, even MCF-7 cells from a single cell bank batch can exhibit heterogeneity: previous work in our lab at the Center for Alternatives to Animal Testing showed that MCF-7 cells coming from the same ATCC lot still displayed marked differences in cellular and phenotypic characteristics, such as proliferation, and expression of estrogen-related genes that escaped routine cell line authentication techniques (Kleensang et al., 2016). A more recent study on MCF-7 also shows variations in expression of reference genes among sub-clones of this cell line (Jain et al., 2020).

In this study, we used large-scale data analysis to examine the similarities and differences between MCF-7—a cell line belonging to the luminal A molecular subtype (Dai et al., 2017)—and invasive breast cancer tissues including four subtypes—luminal A, luminal B, HER2-enriched, and basal-like (Cancer Genome Atlas Network, 2012). To our knowledge, this is one of only a few studies that use network analysis to compare an immortalized cancer cell line to human cancer tissues. The bioinformatics pipeline established in this study was made available and can potentially be applied to similar analysis between cell lines and their corresponding tissues in humans.

## Materials and Methods

### Data

*MCF-7 ARCHS4 data set*. Gene expression level RNA-seq data of the human adenocarcinoma cell line MCF-7 was obtained from the ARCHS4 database (All RNA-seq and ChIP-seq Sample and Signature Search). For detailed description of data processing workflow, readers are invited to read the ARCHS4 article (Lachmann et al., 2018). Briefly, raw RNA-seq data was collected from Gene Expression Omnibus (GEO) by the authors of ARCHS4, aligned to the reference genome, mapped to the gene level, and uploaded to the ARCHS4 database. The MCF-7 data set contained 1032 samples from 107 GEO series. Gene expression data was downloaded as an expression matrix using the R script provided by ARCHS4 and was log_2_-transformed. Since the data set came from multiple experimental series, data sets were checked for batch effects using *Combat* (Johnson et al., 2007) before downstream analysis.

*MCF-7 GSE50705 data set.* RNA microarray data were downloaded from Gene Expression Omnibus (GEO) (Shioda et al., 2013). In the original study, MCF-7 cells were treated with various concentrations of natural and xenobiotic estrogens. We extracted samples treated for 48 h with the steroid hormone 17β-estradiol (*n* = 88), converted probes to gene symbols, and removed probes that were matched to multiple gene names.

*BRCA data set*. Pre-processed, RSEM-normalized Level 3 RNA-seq data of breast invasive ductal carcinoma tissues from The Cancer Genome Atlas was downloaded from FireBrowse. The data set included 1,212 human tissue samples.

**FIGURE 1:**
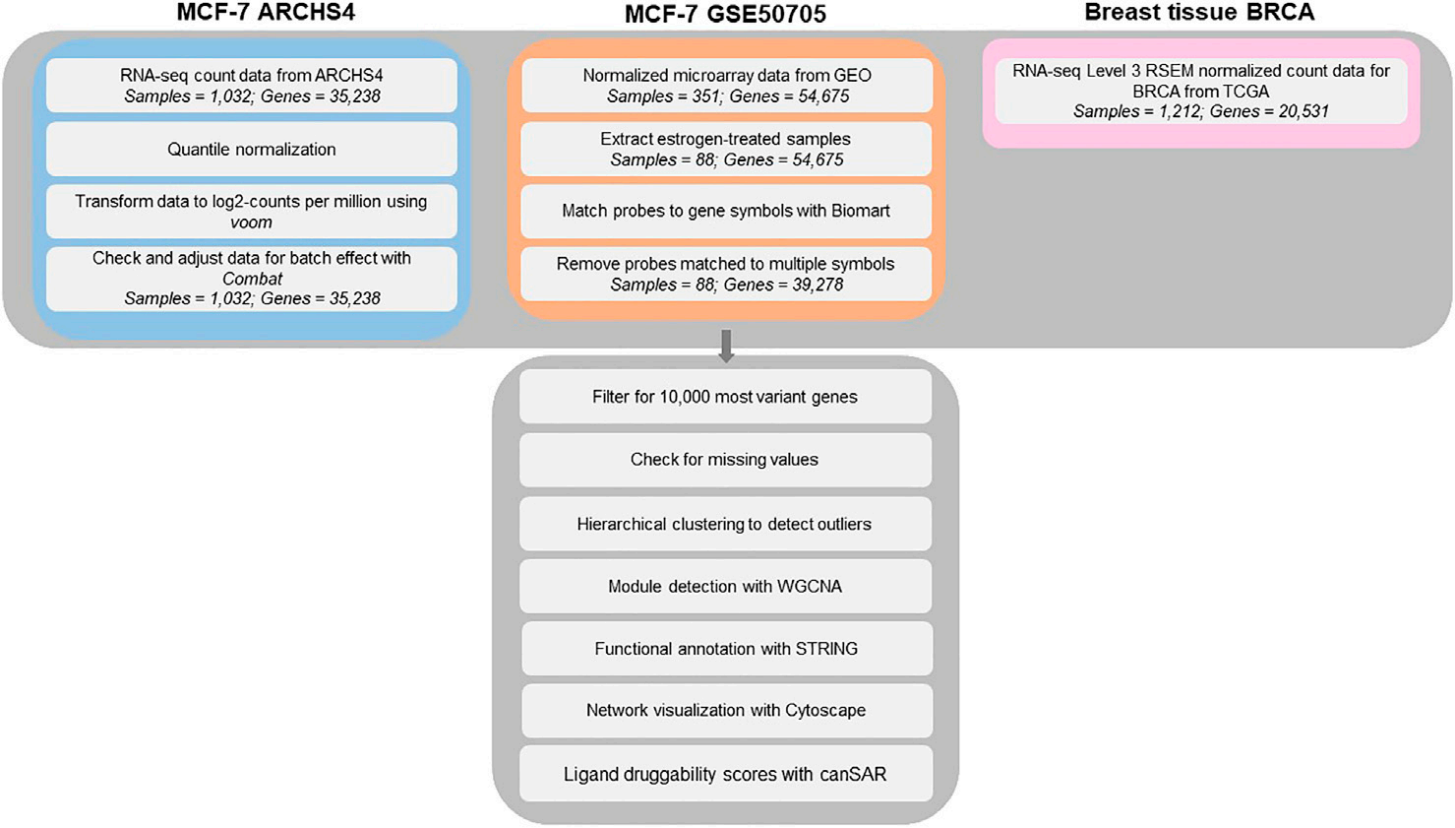
Analysis workflow for the MCF-7 ARCHS4, MCF-7 GSE50705 and BRCA data sets.

For all three MCF-7 and BRCA data sets, samples were checked for outliers using hierarchical clustering, as well as missing values using the *goodSamplesGenes* function in the Weighted Correlation Network Analysis (WGCNA) package. No obvious outliers and missing values were found. Before constructing the co-expression networks for the MCF-7 and BRCA data sets, genes were filtered for the top 10,000 mostly highly variant genes using median absolute deviation (MAD) to exclude the large fraction of genes that are expressed at low level, as two genes with low variance would result in high correlation that would not be biologically meaningful. The resulting gene expression matrices were then analyzed with the WGCNA approach, a popular network analysis algorithm. The analysis workflow for this study can be viewed in Figure 1.

### Weighted Gene Co-expression Network Analysis

WGCNA is a systems biology approach that describes the correlation among genes (Langfelder and Horvath, 2008). It uses network language to describe the pairwise correlation between genes in a data set, based on the assumption that genes with similar expression levels tend to belong to similar pathways. Rather than using a hard threshold for the co-expression similarity *s*_*ij*_, which does not reflect the continuous property of gene expression levels and may lead to loss of information, WGCNA uses a soft threshold approach. It raises the co-expression similarity *s*_*ij*_ to a power *β* (*β* ≥ 1) to obtain the adjacency matrix a_ij_, allowing the adjacency to having continuous values between 0 and 1:

$$a_{ij}=s_{ij}^{\beta }$$

We set *β* = 5 for ARCHS4, *β* = 7 for GSE50705, and *β* = 6 for BRCA based on the scale-free topology criterion (Supplementary Figure S1). After network construction, modules in each data set were detected using hierarchical clustering implemented through the function *blockwiseModules*, with the parameter *minModuleSize* set to 100, 80, and 100 for ARCHS4, GSE50705 and BRCA respectively.

### Functional Annotation

Modules detected by WGCNA can have true biological meaning or can be results of noises in the data, such as sample contamination, technical artifacts, or experimental design. Therefore, we performed functional enrichment of biological processes for genes in each module to identify modules with biological meaning. We used the package *STRINGdb* which provides an R interface to the STRING protein-protein interactions database. The annotation was adjusted for *Homo sapiens* background. Enrichment *p*-values were calculated based on over-representation analysis using hypergeometric tests and were adjusted for multiple hypothesis testing with Benjamini-Hochberg procedure (Szklarczyk et al., 2019).

### Data Visualization

**FIGURE 2:**
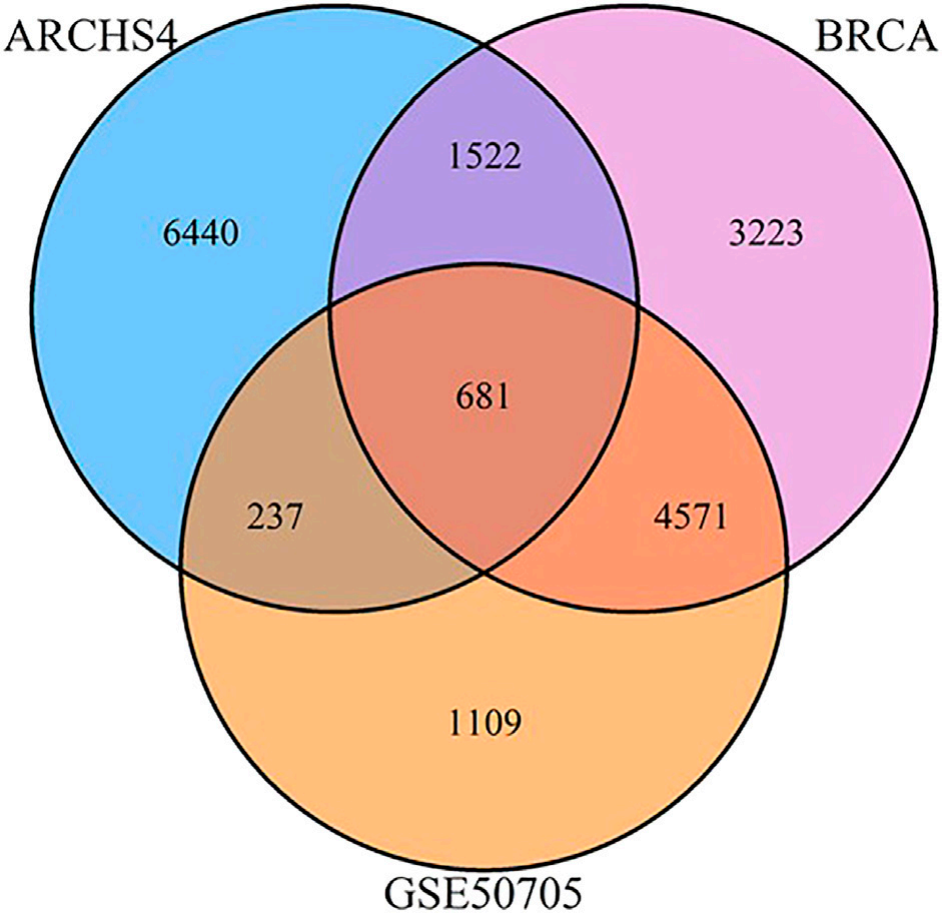
Venn diagram of shared and unique genes between MCF‐7 ARCHS4, GSE50705, and BRCA data sets.

**FIGURE 3:**
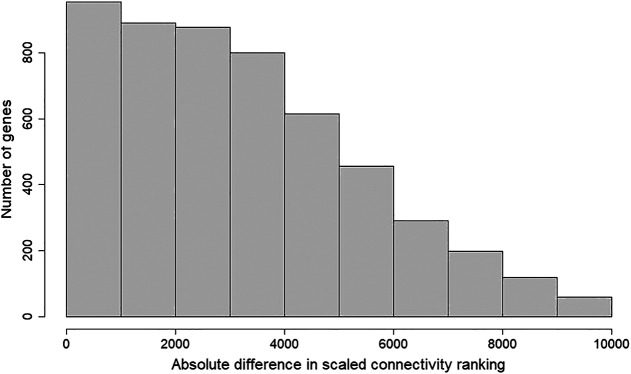
Histogram of absolute difference in scaled connectivity ranking between GSE50705 and BRCA. The scaled connectivity scores obtained with the *fundamentalNetworkConcepts* function from WGCNA was ranked, and the absolute difference between GSE50705 and BRCA were calculated.

**FIGURE 4:**
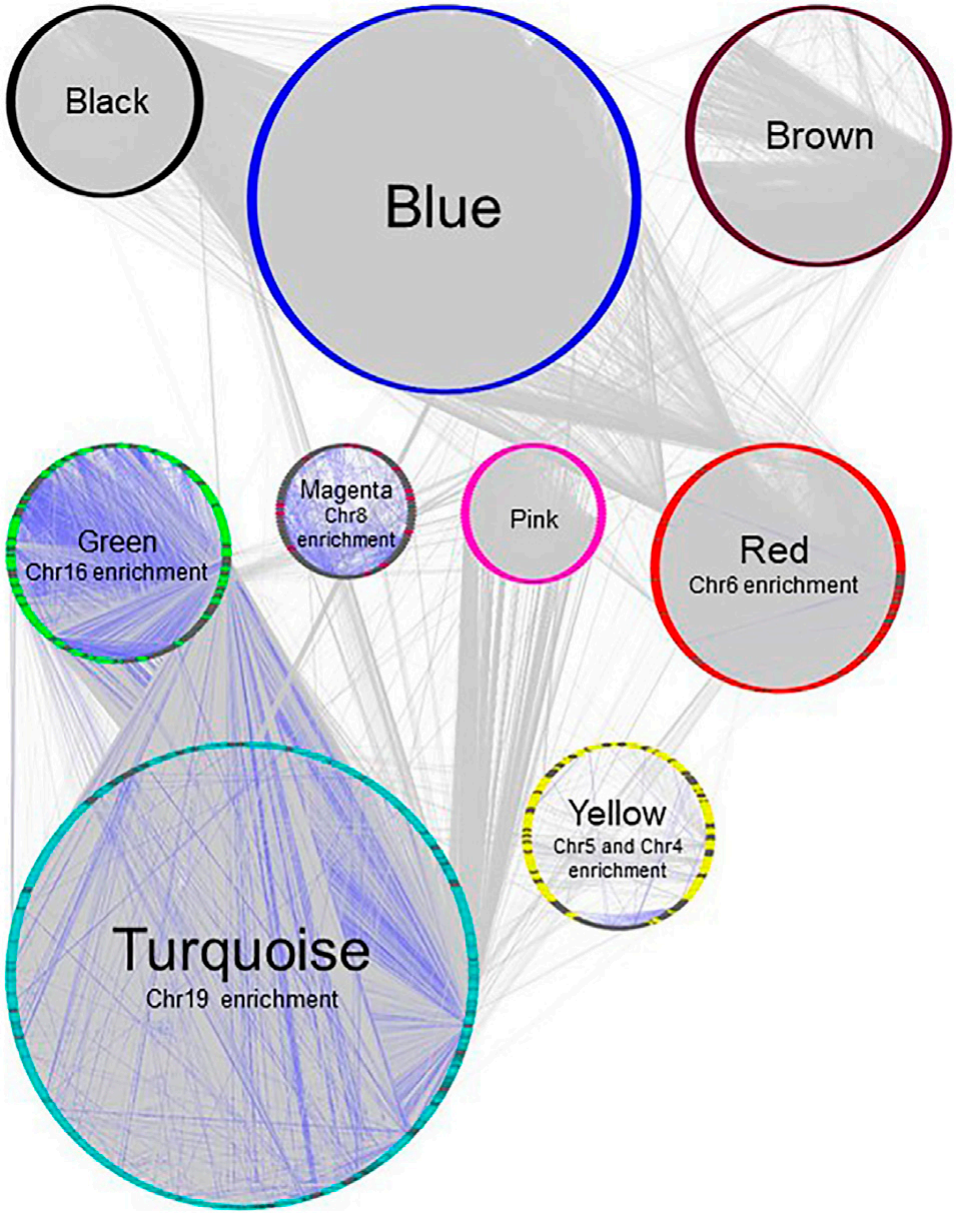
Gene network of BRCA data set, plotted with Group Attributes Layout in Cytoscape. Module membership was determined using WGCNA. All modules except the grey module containing unassigned genes were exported to Cytoscape using the *exportNetworkToCytoscape* function, with a threshold of 0.05. Node color = module color. Genes associated with chromosome enrichment with an adjust-*p* value ≪ 0.05 from Enrichr are highlighted in dark grey. Purple edges indicate interactions between genes enriched for chromosomes.

The modules of interest were visualized with the network visualization software Cytoscape version 3.7.0 (Shannon et al., 2003). For Figures 2 and 3, networks were plotted with Group Attributes Layout in Cytoscape to highlight gene module membership. For Figure 4, the network was plotted with Prefuse Force Directed Layout. Node color was correlated with the number of gene PubMed publications, obtained by querying the Entrez IDs to obtain a raw count of PMIDs on the PubMed database, as described in our previous publication (Maertens et al., 2020).

### Scaled Connectivity

The scaled connectivity for each gene in the MCF-7 and BRCA networks were calculated from the adjacency matrices using the function *fundamentalNetworkConcepts* from the WGCNA package. Scaled connectivity is calculated as *K = Connectivity/*max*(Connectivity)*. Full tables of scaled connectivity of 10,000 most variant genes in GSE50705 and BRCA are available in Supplementary Table 1A,B.

### Ligand-Based Druggability

**FIGURE 5:**
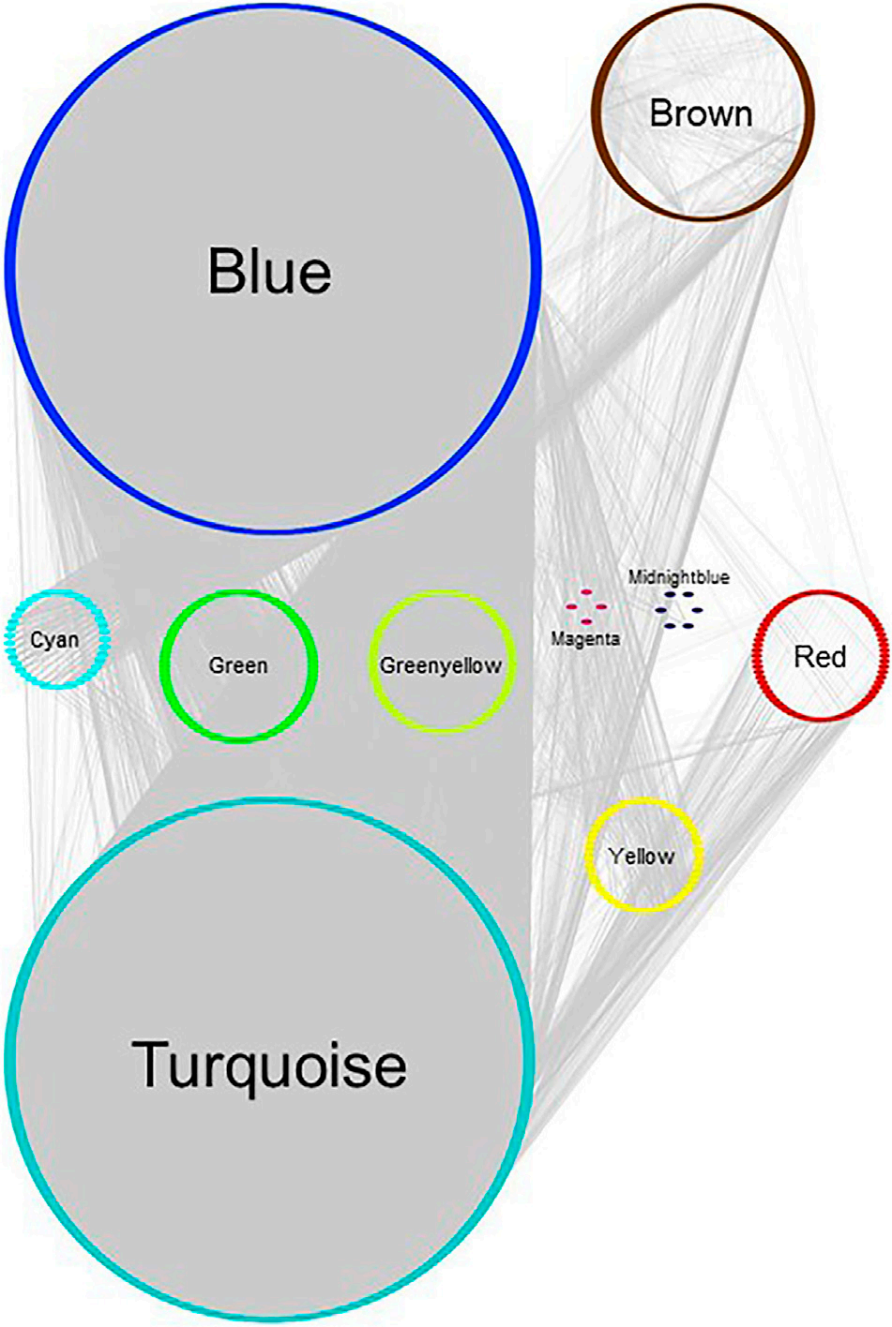
Gene network of MCF‐7 GSE50705 estrogen-treated data set, plotted with Group Attributes Layout in Cytoscape. Module membership was determined using WGCNA. All modules except the grey module were exported to Cytoscape using the *exportNetworkToCytoscape* function, with a threshold of 0.2. Node color = module color. There was no gene significantly enriched for chromosomes in Enrichr.

The ligand druggability scores for the 10,000 most variant genes in the MCF-7 and BRCA data sets were queried using the Protein Annotation Tool from the canSAR knowledgebase. The canSAR database integrates genomic information, structural biology, and properties of compounds to estimate likely “druggability” of chemicals (Tym et al., 2016; Coker et al., 2019). Ligand-based druggability is calculated by looking at the small molecule compounds that have been tested against the protein or its homologues. The ligand-based druggability score for each protein was calculated based on ligand efficiency, med-chem friendliness, and molecular weight of these compounds. Top 30 genes with highest positive ligand druggability scores were selected for each data set for Figure 5. Full tables of ligand druggability for the 10,000 genes in GSE50705 and BRCA are available in Supplementary Table S2A,B.

## Results

### Minimal Overlapping Genes Between Michigan Cancer Foundation-7 and Human Breast Tissues

**FIGURE 6:**
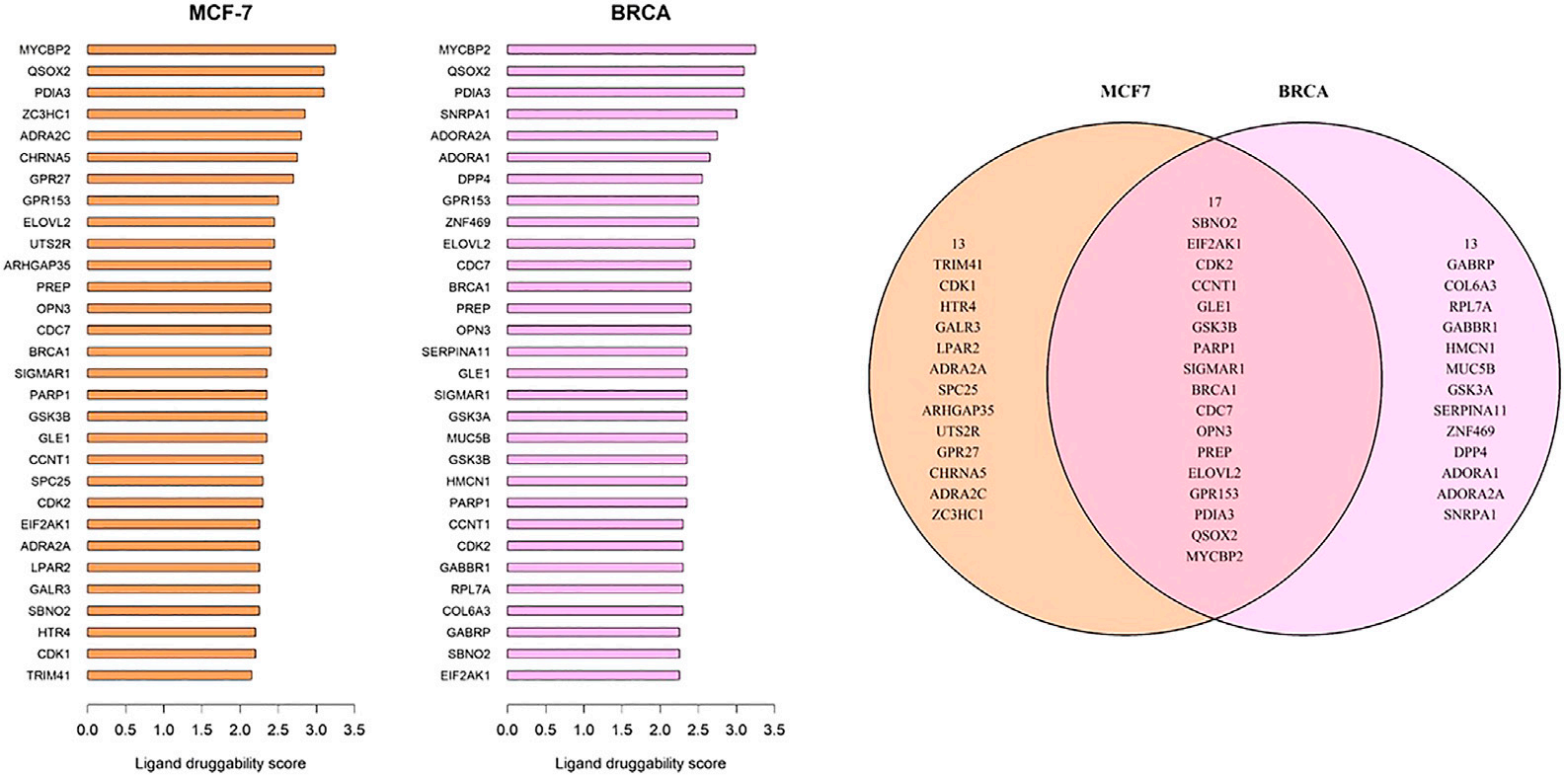
Genes with top ligand druggability scores for **(A)** GSE50705 and **(B)** BRCA **(C)** Among the top 30 genes, 17 genes are overlapping.

We selected three data sets based on human breast cancer tissues: 1) the TCGA data set of invasive breast cancer biopsies (henceforth BRCA), which has the advantage of reflecting human *in vivo* samples, although biopsies by their nature include a mix of different tissues 2) the ARCHS4 collection of MCF-7 samples, which is an attempt to massively mine publicly available RNA-seq experiments, and consists of 1032 samples combined from GEO, and 3) a smaller study of MCF-7 cells exposed to estrogen in a dose response curve. As the data sets involve a range of different technologies, preprocessing strategies, and in the case of ARCHS4, potentially many different biological conditions, we began with the basic initial step of reducing the gene expression set to the top 10,000 most variant genes, to eliminate genes that were minimally or inconsistently expressed and would therefore confound the use of a correlation-based approach. Surprisingly, even this initial step indicated minimal conservation of gene expression signatures - only 681 genes were conserved amongst the three datasets, and of the top 10,0000 genes from the ARCHS4 data set, fully 6,440 were unique to that data set (Figure 6).

In the case of the genes found in all three data sets, annotation analysis revealed that they were enriched for genes annotated to mitotic cell cycle (adjusted-*p* value = 1.08E-21), regulation of cell migration (adjusted *p* = 1.89E-18), and response to endogenous stimulus (adjusted-*p* value = 2.33E-18) (Supplementary Table S3), suggesting that of the highly expressed genes, the common genes are likely annotated to fundamental cell processes.

In order to understand how and why the data sets diverged even at this fundamental level, we explored the genes that were unique to each data set. For the BRCA data set, we suspected that one cause of the difference was likely the fact that cancer biopsies always reflect a mixture of cell-types and typically have a significant component of immune infiltration. Our data support this to a limited extent: genes unique to BRCA were enriched for immune-related GO annotations, such as regulation of immune response (adjusted-*p* value = 0.002023) and regulation of innate immune response (adjusted-*p* value = 0.007635) (Supplementary Table S3). In addition, within genes mapped to cell types via the Human Gene Atlas, there was a modest level of enrichment for immune-cell related genes (Supplementary Table S3).

More striking, however, was a marked presence of ribosomal subunit genes unique to the BRCA data set, annotated via STRING as ribosome biogenesis (adjusted *p*-value = 1.81E-08) (Supplementary Table S3), and Bioplanet as Cytoplasmic ribosomal proteins (adjusted-*p* value = 1.61E-10). While these ribosomal subunit genes are ubiquitously expressed in most breast cancer cell lines as well as most tissues (Ebright et al., 2020), they were in neither the MCF7-derived GSE50705 data set nor the ARCHS4 data set, likely owing to some extent to the chip design for the GSE50705, and the high noise level in ARCSH4. Within the BRCA data set, several ribosomal proteins showed a high level of patient-to-patient variation (Supplementary Figure S2A). Of the top most variant ribosomal proteins within the BRCA data set, only RPS3 was also in the GSE50705 data, and a much narrower dynamic range (Supplementary Figure S2B). Strong ribosomal signatures in a subset of circulating tumor cells have been associated with poor clinical outcomes in breast cancer patients (Ebright et al., 2020), and it seems likely that MCF-7 cells may not capture the effects of the variation in ribosomal protein expression patterns.

The genes unique to the MCF-7 GSE50705 estrogen dose-response curve were enriched for genes related to non-coding RNA processing (adjusted-*p* value = 6.64E-09) and mitochondrial respiratory chain complex IV biogenesis (adjusted-*p* value = 1.77E-07). Meanwhile, the large set of genes unique to the ARCHS4 dataset are most significantly enriched for cell-cell signaling (adjusted-*p* value = 2.23E-59), synaptic transmission (adjusted-*p* value = 8.52E-56), and ion transport (adjusted-*p* value = 9.11E-41) (Supplementary Table S3).

As breast cancer cell lines, it is surprising that genes unique to ARCHS4 MCF-7 cells are enriched for generation of neurons (adjusted *p*-value 5.98E-21) — a process unique to neuronal cells. In addition, there were some genes annotated to the meiotic chromosome segregation in this data set and even a few Y chromosome genes (Supplementary Table S3). This can be caused by artifacts of annotations data or possibly contamination of other cell lines during experimental design of GEO studies or inclusion of non-MCF7 samples during data mining for ARCHS4. Overall, we observed a higher degree of similarity between GSE50705 and BRCA (5252 overlapping genes) than between ARCHS4 and BRCA (2203 overlapping genes).

### Network Signatures Indicate Substantial Differences Between Data sets

In order to investigate similarities and differences between the data sets at a more intricate level, we used WGCNA for the 10,000 most variant genes in each data set to assign the genes to functional modules and see if, broadly speaking, interactions amongst genes were conserved. WGCNA uses correlations amongst gene expressions and groups genes in an unsupervised way to determine potential interactions. In keeping with our previous studies (Maertens et al., 2018; Maertens et al., 2020), the modules produced by WGCNA were input into STRING for biological annotations to verify whether WGCNA had produced modules of genes that were known to interact and were enriched for annotations.

For both the BRCA and the GSE50705 data sets, the modules were enriched for known protein interactions in STRING as well as highly significant adjusted *p*-values for GO Biological Processes, indicating that for most of the genes, WGCNA indeed clustered genes with similar biological functions and on the same pathways. However, the modules of the ARCHS4 dataset were unsatisfactory: most genes could not be classified into modules and ended up in the grey module for unassigned genes (Supplementary Figure S3A). These modules were also small and lacked distinguishing enrichments in STRING (Supplementary Table S4A). Due to the lack of meaningful biological signals in ARCHS4, we decided to focus subsequent analyses on the GSE50705 and BRCA data sets.

Annotations by modules in the GSE50705 and BRCA data sets indicated that the BRCA data set had a red module (Supplementary Table S4B) that was enriched for genes annotated as immune-related or cytokine-response biological processes. As expected, the immune component was not present in the GSE50705 data (Supplementary Table S4C). Our finding is supported by another study showing consistent upregulation of immune processes in primary tumors, possibly a result of immune infiltration in tumor tissues that is not present in cell lines (Yu et al., 2019).

Interestingly, even modules annotated for the same pathways in the two data sets varied substantially in their gene constituents: the largest module annotated for cell cycle processes in the BRCA data set (brown module, 1013 genes) only has 354 genes overlapping with its counterpart in the MCF-7 GS550705 data set (turquoize module, 2244 genes).

To determine whether the MCF-7 and BRCA data sets differ regarding network topology, we calculated the scaled connectivity, a metric for gene significance in a network, which asks if the gene is acting as a hub, or highly connected gene. We observed substantial difference between the two data sets: the average mean absolute difference between scaled connectivity was 3,223 and a small cluster of genes had a difference in scaled connectivity greater than 9,000, in each case ranking significantly higher in the MCF-7 dataset than the TCGA (Figure 5). The genes with the highest scaled connectivity in MCF-7 *vs*. TCGA showed relatively rare over- or under-expression within the BRCA data set, in each case with transcriptional perturbations less than 10 percent (Table 1, Supplementary Figure S4). The relatively high connectivity in MCF-7 may reflect a combination of the lineage of MCF-7 (perhaps a cancer type that over-expressed one or more of these genes), or the result of cellular instability and the evolution of MCF-7 over time.

**TABLE 1:** Top 10 genes with highest difference in scaled connectivity ranking between GSE50705 and BRCA.

GeneSymbol	Scaled connectivity in MCF7	Rank in MCF7	Scaled connectivity in BRCA	Rank in BRCA	Absolute ranking difference
SUSD2	0.787126168	68	6.48E-05	9995	9927
CLU	0.878769987	16	0.000544507	9933	9917
BCAR3	0.78900237	67	0.001091298	9861	9794
TMPRSS3	0.767847573	82	0.000989588	9875	9793
OLFM1	0.70620407	181	0.000370951	9960	9779
SLC24A3	0.748824023	108	0.000982308	9877	9769
DEGS1	0.72618254	148	0.00099857	9874	9726
NPY1R	0.752667373	103	0.001839083	9775	9672
PLK2	0.722418194	156	0.001623592	9802	9646
CYP2J2	0.674845098	238	0.000986705	9876	9638

### Breast Invasive Ductal Carcinoma Data Modules Contain Substantially More Cis-Regulated Genes Compared to GSE50705

In our previous study using WGCNA to analyze genes for functional assignment (Maertens et al., 2020), we reported that some modules were significantly enriched for genes on a single chromosome and that these modules tended to have no statistically significant enrichments for protein-protein interactions (PPI) or functional enrichments. Others have similarly reported significant clustering of genes based on intra-chromosomal distance and this feature has been demonstrated to be specific for different phenotypes (Garcia-Cortes et al., 2020). We found that the BRCA data had several modules that, although enriched for both PPI interactions and functional enrichments, had a statistically significant enrichment for genes on a single chromosome (Figure 2), likely reflecting increased co-expression from neighboring genes that results from a disruption in the regulatory elements that control gene transcription. Interestingly, this is not true of the MCF-7 sample - no module was significantly enriched for any one chromosome - despite the fact that it originates from a cancer cell line (Figure 3). However, it should be noted that the difference may simply be due to the greater dynamic range of RNA-sequencing compared to microarray.

### Potential Drug Targets Missed by Using Michigan Cancer Foundation-7

As MCF-7 is often used for drug discovery research, we wanted to explore whether any potentially druggable candidates would be missed. To identify potential drug targets, we used the CanSAR database, which is a protein structure-based model that predicts ligand-based druggability scores based on the predicted cavities of over 144,000 proteins (Coker et al., 2019). While there was some overlap between the top-ranked druggable genes, there were several genes that would likely have been missed if MCF-7 were exclusively used for protein targets. However, it should be kept in mind that this approach is merely looking for potential candidates based on protein accessibility, not cancer biology, and there was no available information about classes of drugs. Therefore, our results merely suggest that it might be useful to look outside the MCF-7 model when screening for cancer drug targets.

As an example, of the genes that ranked highly for ligand-based druggability, CCNT1 has the 23rd highest score in the BRCA data set and was ranked in 277th in scaled connectivity in this data set yet ranked 5,824th in the GSE50705 data set. Altered CCNT1 expression (defined as z-score > +/−1.5) is not significantly associated with any mutations or copy number variations. Interestingly, altered expression is significantly associated with race, being more common in African Americans compared to Whites or Asian Americans (Supplementary Figure S5) - while the reason for this may range from SNP polymorphisms to different environmental exposures amongst populations, it does indicate one intrinsic short-coming of MCF-7 cells: they were isolated from a White female (Comsa et al., 2015), and therefore a model of breast cancer based on this tissue type alone will miss much of the molecular diversity of breast cancer in a population with mixed genetic backgrounds, diverse environmental exposures and clinical histories (Makki, 2015; Koual et al., 2020).

**FIGURE 7:**
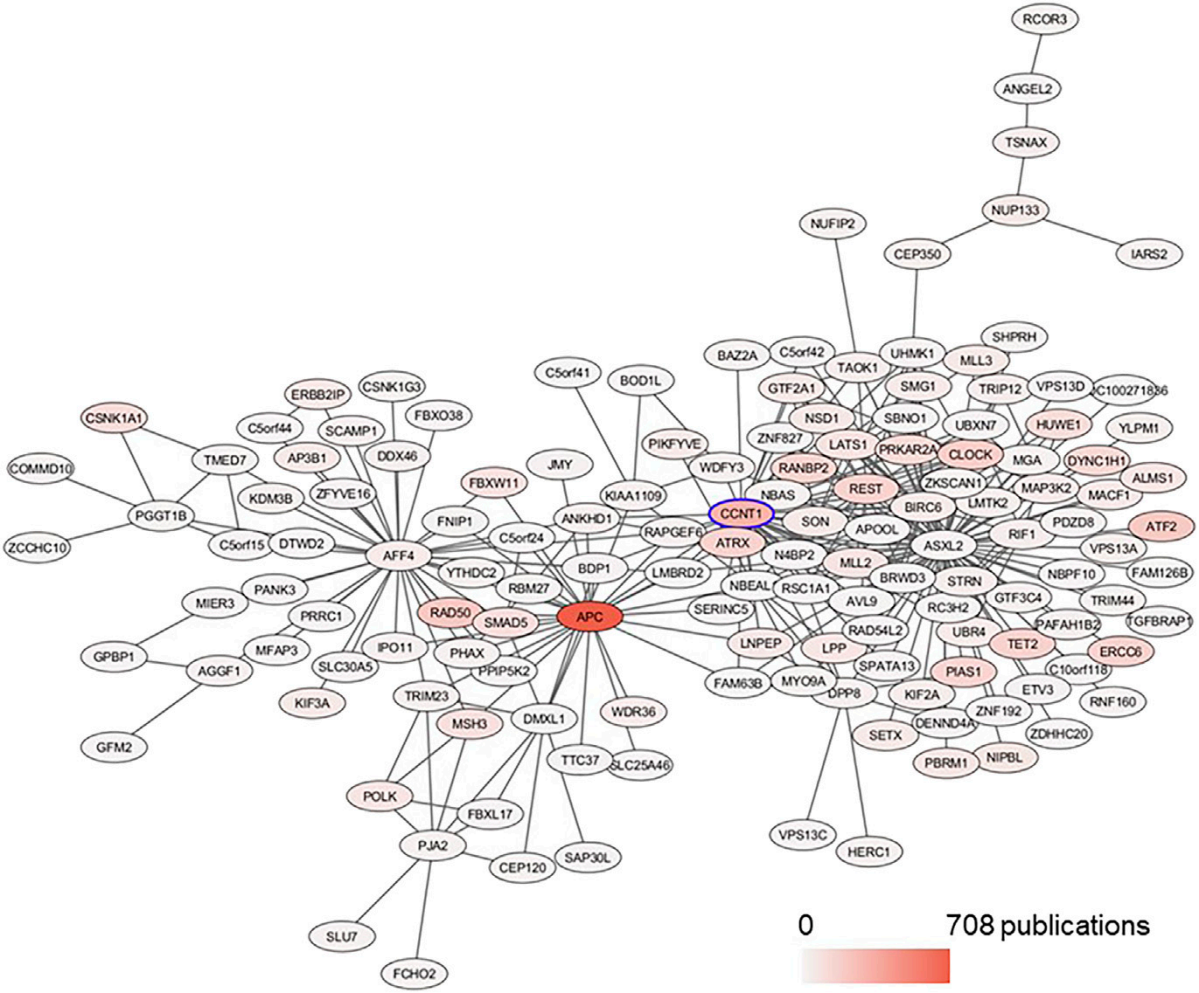
Subnetwork of CCNT1 in the BRCA data set plotted with Prefuse Force Directed Layout in Cytoscape. From the module yellow in BRCA, the first neighbors of CCNT1 were selected. The color of the node corresponds to the number of PubMed publications associated with the genes.

In the BRCA data set, CCNT1 was located in the yellow module enriched for several biological processes (intracellular transport - adjusted *p* value 4.63E-12, protein modification by small protein conjugation or removal - adjusted *p* value 4.63E-12, and protein transport - adjusted *p* value 2.11E-09) as well as Chromosome 4 (adjusted *p* value 0.0009227) and Chromosome 5 (adjusted *p* value 7.435E-20) enrichments (Supplementary Table S5). In order to predict the most likely trans-activated co-expressed genes, we looked at the top 50 genes correlated with CCNT1 expression by Spearman rank correlation (coefficient of correlation > 0.789; *q*-value < *p* = 6.27e-235). While these genes were enriched for known protein-protein interactions via STRING (*p*-value 0.000268), many of the genes were unconnected, and the only significant GO annotations were each based on a maximum of three genes - likely because many of these genes have minimal literature (Figure 7), and 31 of the genes were “unclassified” in GO SLIM Biological Process and therefore invisible in any annotation-based approach.

Using the FANTOM EdgeExressDB (Lizio et al., 2015; Lizio et al., 2019) to explore potential connections suggested that the coordinated co-expression of many of the genes was possible due to the transcription factor CLOCK, which had a very high correlation with CCNT1 - indeed, the two genes are significantly correlated in every cancer within TCGA, both before and after adjusting for tumor purity. So, while the mechanism of the correlated co-expression is unknown, it is a fairly robust finding within cancer tissues. The Spearman correlation between CCNT1 and CLOCK in the GSE50705 dataset was 0.130 with a *p*-value of 0.2285, and the top 50 candidates based on Spearman rank were shown in Supplementary Table S6.

While CCNT1 is not associated with survival in breast cancer as a whole, low expression is associated with longer survival in luminal A cancer (HR 1.98, *p* = 0.0155); similarly, low CLOCK expression was associated with increased survival in all breast cancer subtypes as well as the luminal A subtype. Like CCNT1, altered CLOCK expression is more common in African Americans.

The ultimate molecular function of CCNT1 and its interactions with CLOCK and the other predicted genes remains elusive, and any potential role in breast cancer is largely unremarked, as only 3 papers within PubMed mention CCNT1 and breast cancer, and in fact only 39 papers mention CCNT1 and cancer. The significance of CLOCK in cancer is better understood as it is thought to be a molecular link between disrupted circadian rhythm and cancer (Trujillo and Muotri, 2018), including breast cancer (Cadenas et al., 2014; Xiao et al., 2014).

## Discussion

Cell lines are often used as models for cancer research, but recent studies have drawn attention to the ways in which cell-lines can introduce artifacts. MCF-7 is not the only cell line that expresses heterogeneity. Other commonly used breast cancer cell lines such as T47D, BT474, and SKBR3 have also been shown to develop chromosomal alterations through cluster analysis (Rondon-Lagos et al., 2014). A recent study on the reproducibility of a perturbational assay in anti-cancer drugs using the human mammary epithelial cell line MCF10A shows variability of findings among five research centers, although it should be noted that the observed variability can be due to multiple biological, experimental and computational factors (Niepel et al., 2019).

One interesting result of this study is the lack of concordance between the ARCHS4 and TCGA data sets. Above and beyond the obvious reasons for differences between *in vivo* cancer tissue and a larger collection of *in vitro* studies with varying experimental conditions, there are likely differences introduced from the data analysis pipelines. Nonetheless, it remains surprising that even at the basic level of sorting by variant genes, there was very little in common with other MCF7-based studies. Correlation based approaches are often used on large data sets to find commonly expressed genes - and this function is built into ARCHS4 - but in this application, the size of the data set did not appear to compensate for the increased noise when it came to teasing out possible interactions.

Our examination of the smaller data set based on MCF-7 cells treated with estradiol and breast cancer tissues shows that although there are some conserved genes between the two networks, the majority of genes were non-overlapping. This issue has been raised in other studies: for instance, the Wellcome Sanger Institute used the CRISPR-cas9 screens on 324 human cell lines to priority gene targets for 30 cancer types, and found 628 priority targets (Behan et al., 2019); however, the vast difference between these cell lines and *in vivo* patient data meant that at least some of the targets predicted by CRISPR-cas9 screens were irrelevant to human cancer biology (Lyu et al., 2020). In our study, the substantial differences in co-expression networks between the MCF-7 cell line and human breast cancer tissues could have many explanations - cell line evolution of the MCF7 cells after multiple generations, as well as the increase in cis-regulated expression in *in vivo* cancer, and technical differences between the transcriptomic approaches. Nonetheless, the heterogeneity among BCRA patients likely contributes to a great deal to the difference. Our study did, like many studies of *in vitro* tumors, underscore that tumors contain multiple cell-types, as evidenced by the presence of a module of immune-related genes.

Similarly, our finding of marked correlations amongst genes based on chromosome distance within the TCGA data set, but not in the GSE data set, suggests that correlation based approaches used on cancer-derived tissues requires caution, as cis-activation (or uncontrolled transcription) will cause markedly strong associations not driven by a common transcription factor binding sites or pathways (Garcia-Cortes et al., 2020), and this complicates any interpretation of the scaled connectivity score or assuming any correlation reflects a specific interaction. The observation of cis-activated genes in the TCGA data set and not in the GSE data set could be due to the difference in dynamic range between RNA-seq and microarray. However, these correlations could also be *biologically* meaningful, potentially caused by pervasive copy number variations within the 19p13 chromosome region in breast cancer tissues, and the increased transcription almost certainly has biological consequences.

There are several limitations in our study: although WGCNA is a powerful bioinformatics method, it can result in false positives and spurious correlations. We addressed this issue by examining the gene modules resulting from the WGCNA algorithm for biologically meaningful annotations. Another limitation is the shortcoming of annotation databases, as mentioned in our previous publication (Maertens et al., 2020). Finally, the difference between MCF-7 and BRCA we observed could also be accounted for by the difference in mRNA sequencing technologies. The MCF-7 GSE50705 data set was measured with the microarray platform Affymetrix Human Genome U133 Plus 2.0 Array, and the BRCA TCGA data set was measured with the RNA seq technology Illumina HiSeq 2000 RNA Sequencing Version 2. RNA-seq is more sensitive than microarray to low-abundance transcripts (Wang et al., 2014), and the concordance between RNA-seq and microarray technologies can vary from low to high (Zhao et al., 2014; Trost et al., 2015).

Nevertheless, our study indicates that both models have limitations: MCF-7 cells lack genetic diversity and are known to have a significant lack of reproducibility; at the same time *in vivo* tumors will have greater cellular heterogeneity and artifacts intrinsic to cancers, such as greater cis-regulation. This is perhaps a useful reminder of the truism that all models are wrong, but some models are useful - and that the models are more useful when we know in what ways they are likely to mislead us.

## Data Availability

The ARCHS4 MCF-7 data set was obtained from the ARCHS4 database. The GSE50705 MCF-7 data set was obtained from the GEO database. The BRCA data set was obtained from the FireBrowse database. R codes for our data analysis are available on GitHub. ARCHS4 database: https://maayanlab.cloud/archs4/data.html GEO database: https://www.ncbi.nlm.nih.gov/geo/query/acc.cgi?acc=GSE50705 FireBrowse database: http://firebrowse.org/?cohort=BRCA&download_dialog=true GitHub: https://github.com/vy-p-tran/Similarities-and-differences-in-gene-expression-networks-between-MCF7-and-BRCA.
