# Supplementary material for: Similarities and Differences in Gene Expression Networks Between the Breast Cancer Cell Line Michigan Cancer Foundation-7 and Invasive Human Breast Cancer Tissues
Source: Front Artif Intell. 2021 May 13;4:674370. doi: 10.3389/frai.2021.674370 (PMC8155268; doi:10.3389/frai.2021.674370)
Supplement: Supplementary file 1 [file DataSheet1.zip › Supplemental Figures and Tables/Supplemental Figure 2b. Expression of genes enriched for ribosomes GSE50705.pdf]

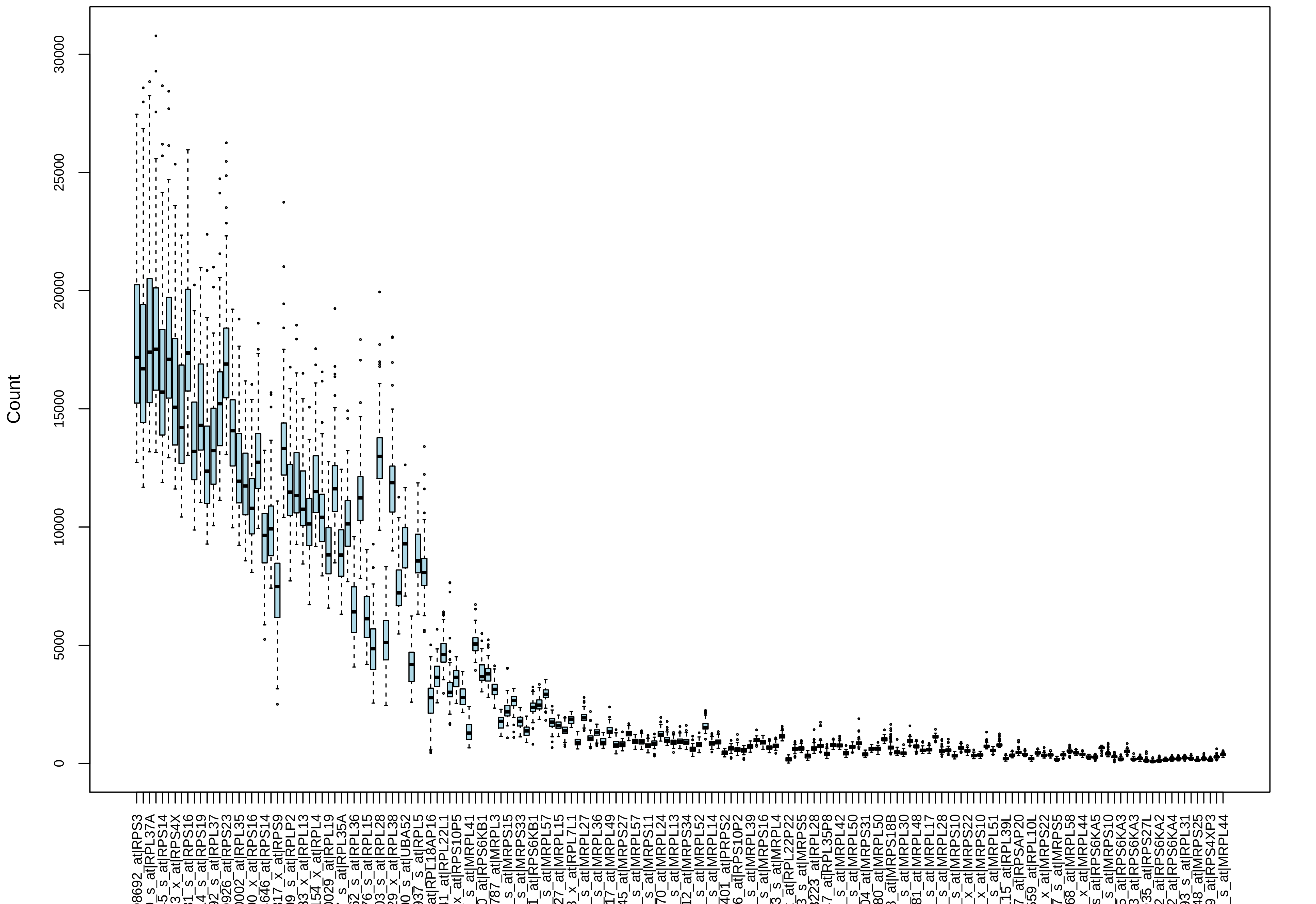
Count
0
5000
10000
15000
20000
25000
30000
