# Supplementary figures and images for: Similarities and Differences in Gene Expression Networks Between the Breast Cancer Cell Line Michigan Cancer Foundation-7 and Invasive Human Breast Cancer Tissues
Source: Front Artif Intell. 2021 May 13;4:674370. doi: 10.3389/frai.2021.674370 (PMC8155268; doi:10.3389/frai.2021.674370)

## Scale independence

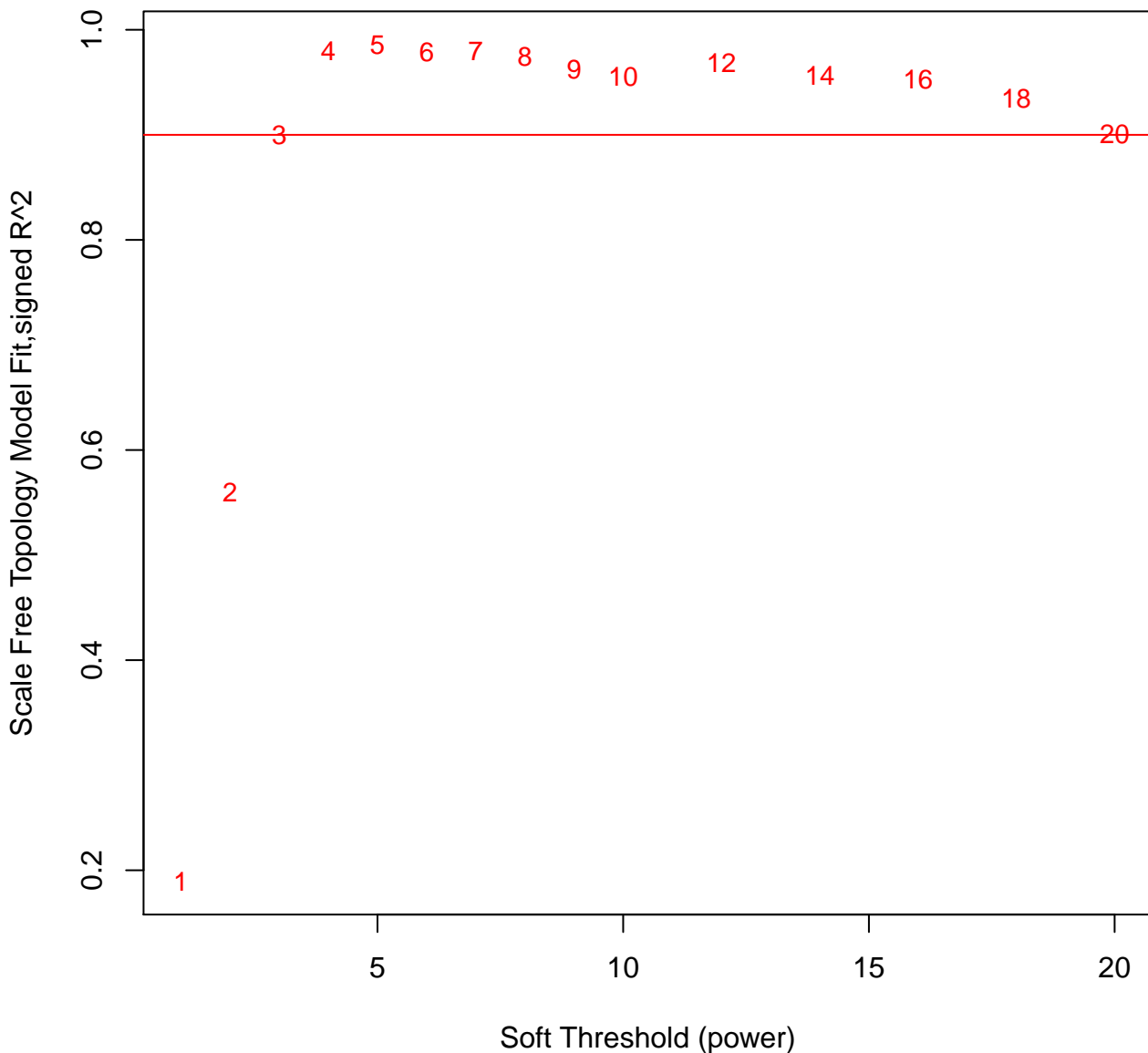

## Mean connectivity

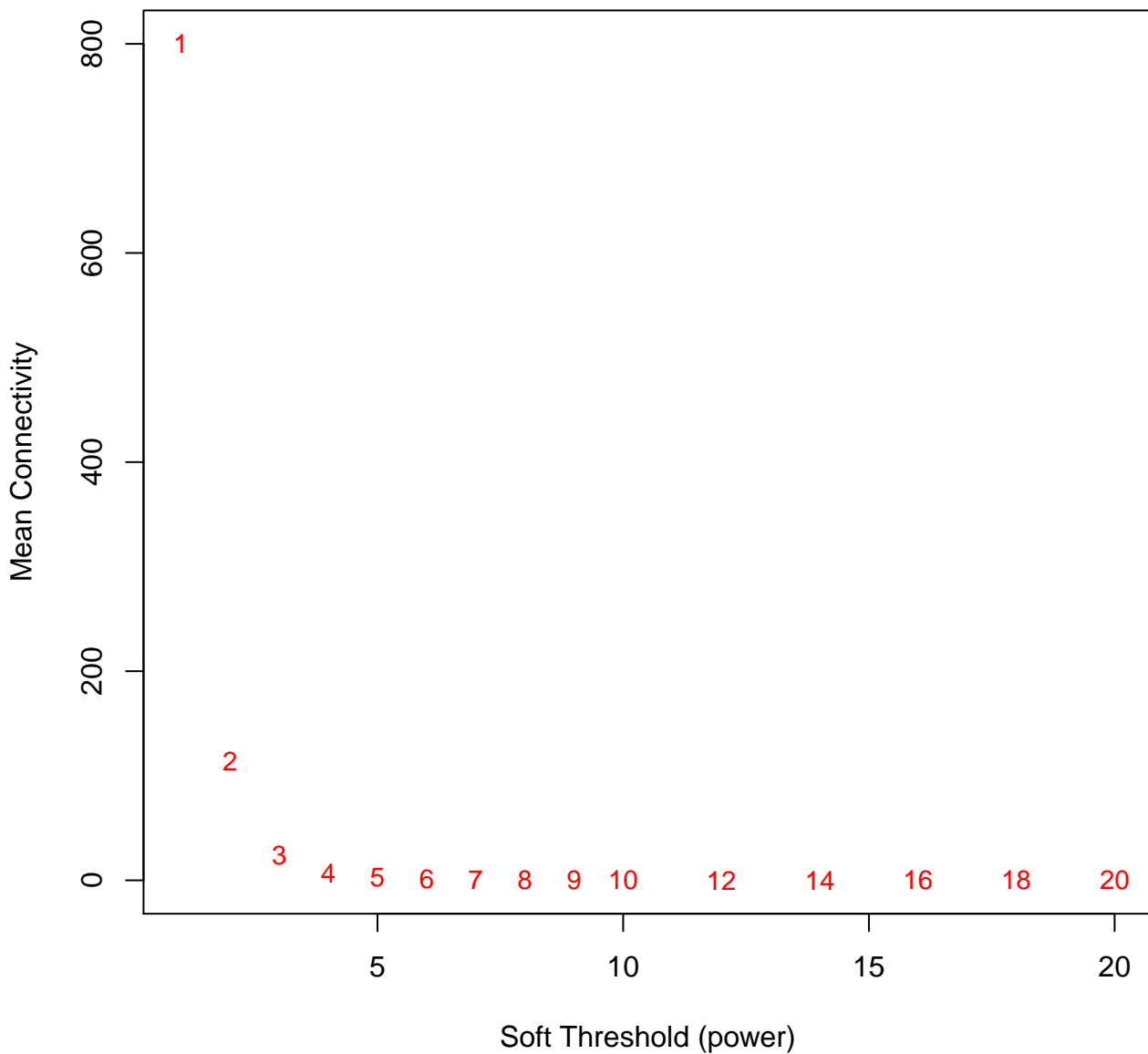

Supplement: Supplementary file 1 [file DataSheet1.zip › Supplemental Figures and Tables/Supplemental figure 1 a and b. Scale independence and Mean connectivitity for ARCHS4.pdf]

## Scale independence

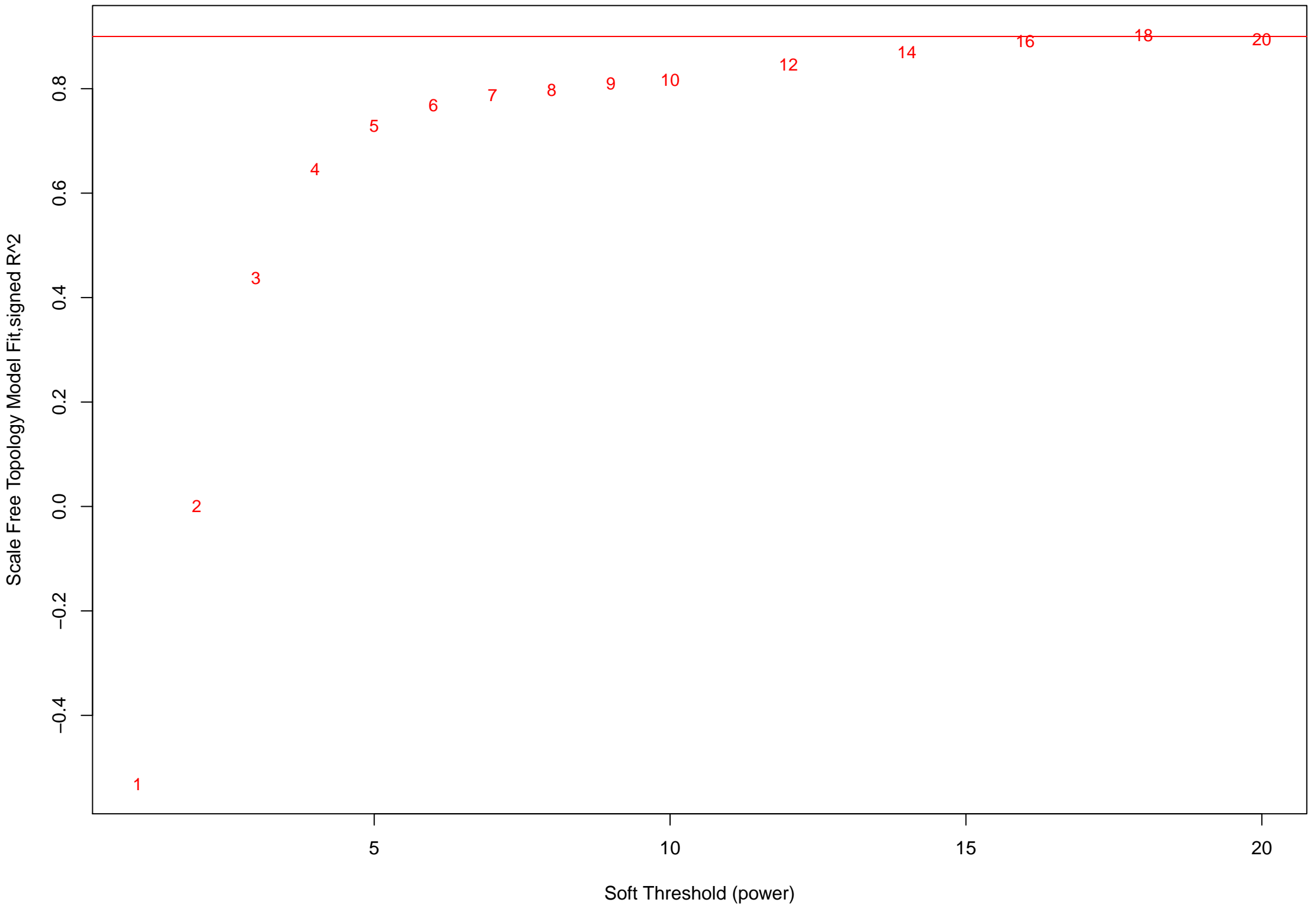

Mean connectivity

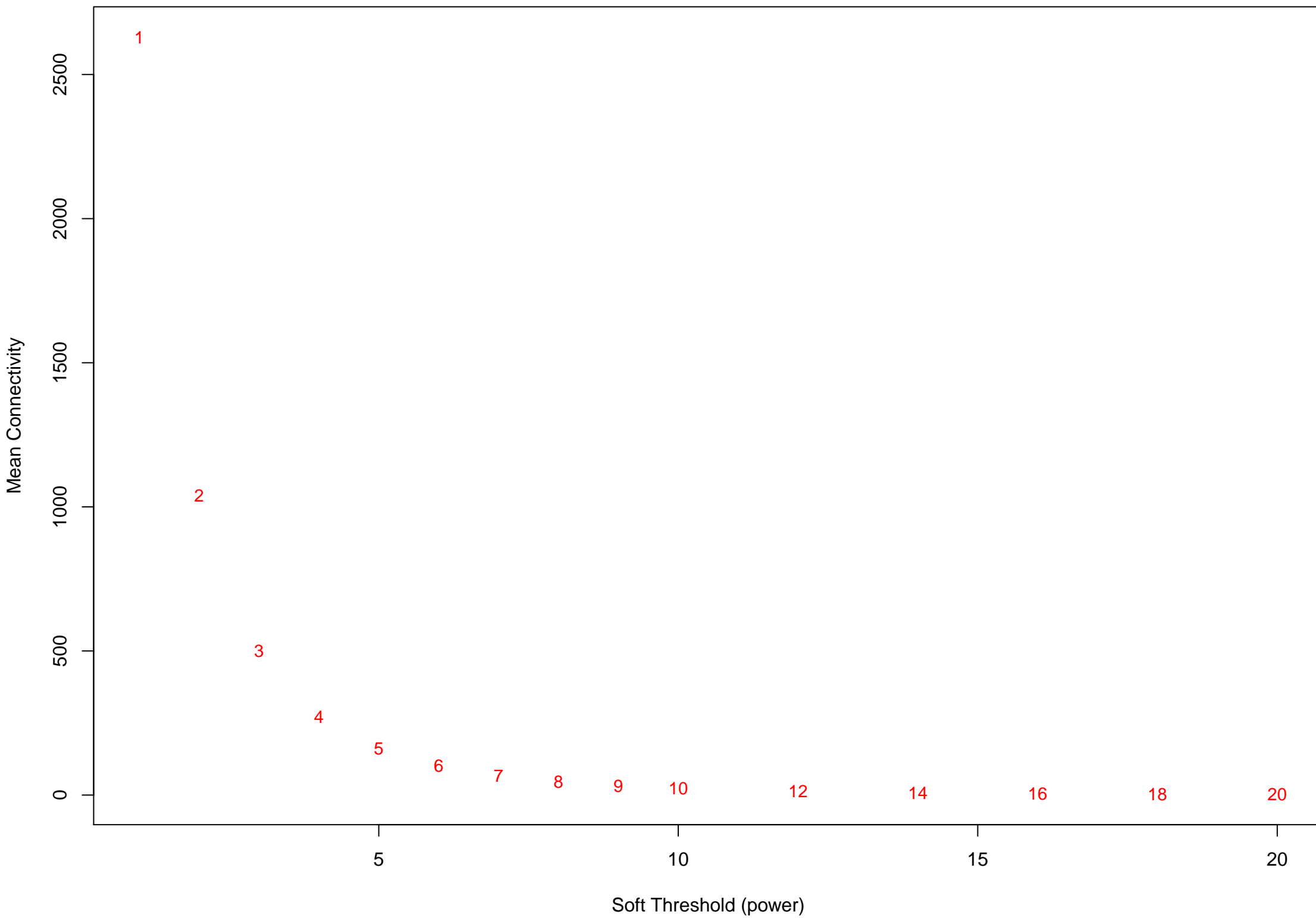

Supplement: Supplementary file 1 [file DataSheet1.zip › Supplemental Figures and Tables/Supplemental Figure 1 c and d. Scale independence and Mean connectivity for GSE50705.pdf]

## Scale independence

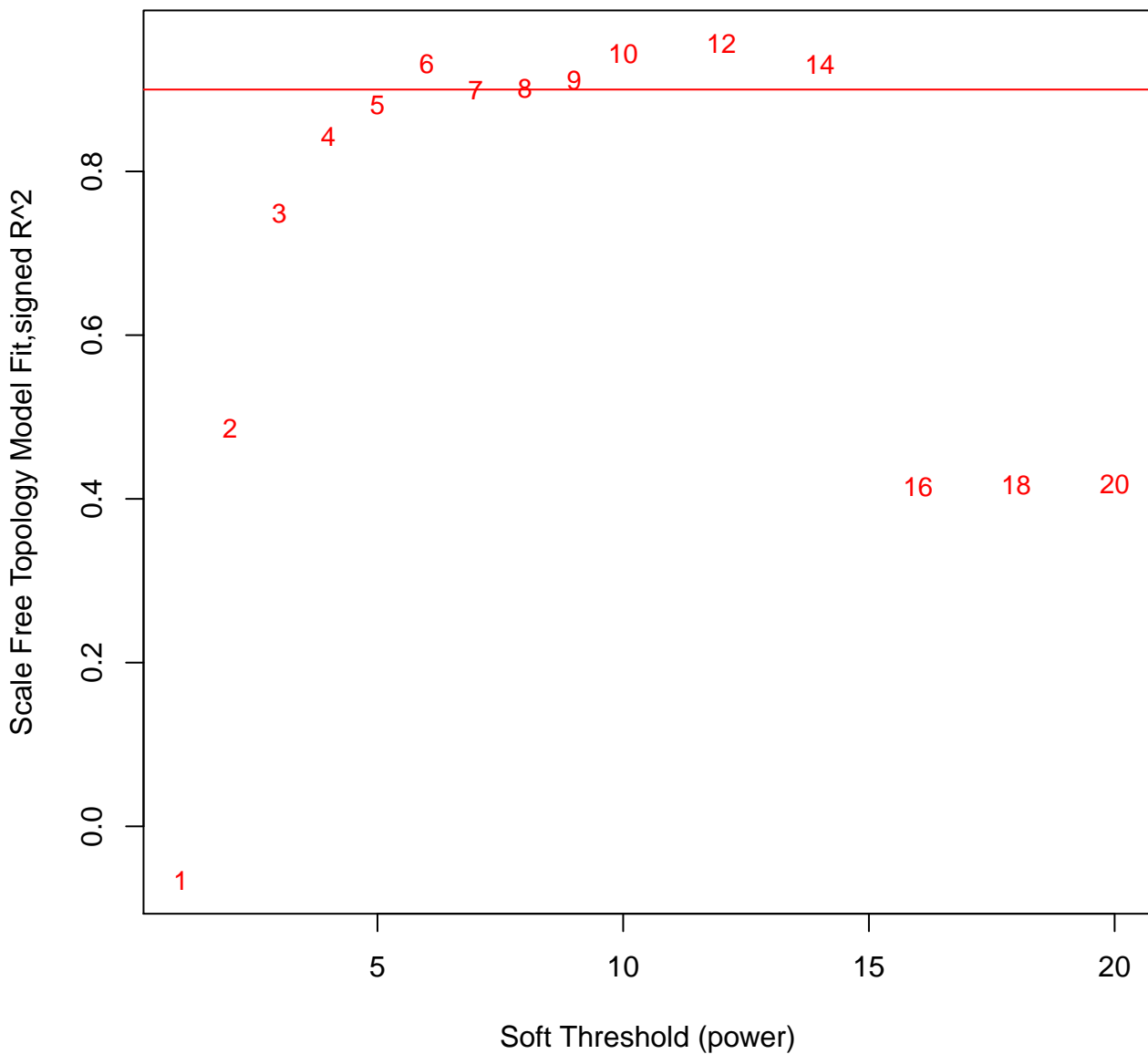

## Mean connectivity

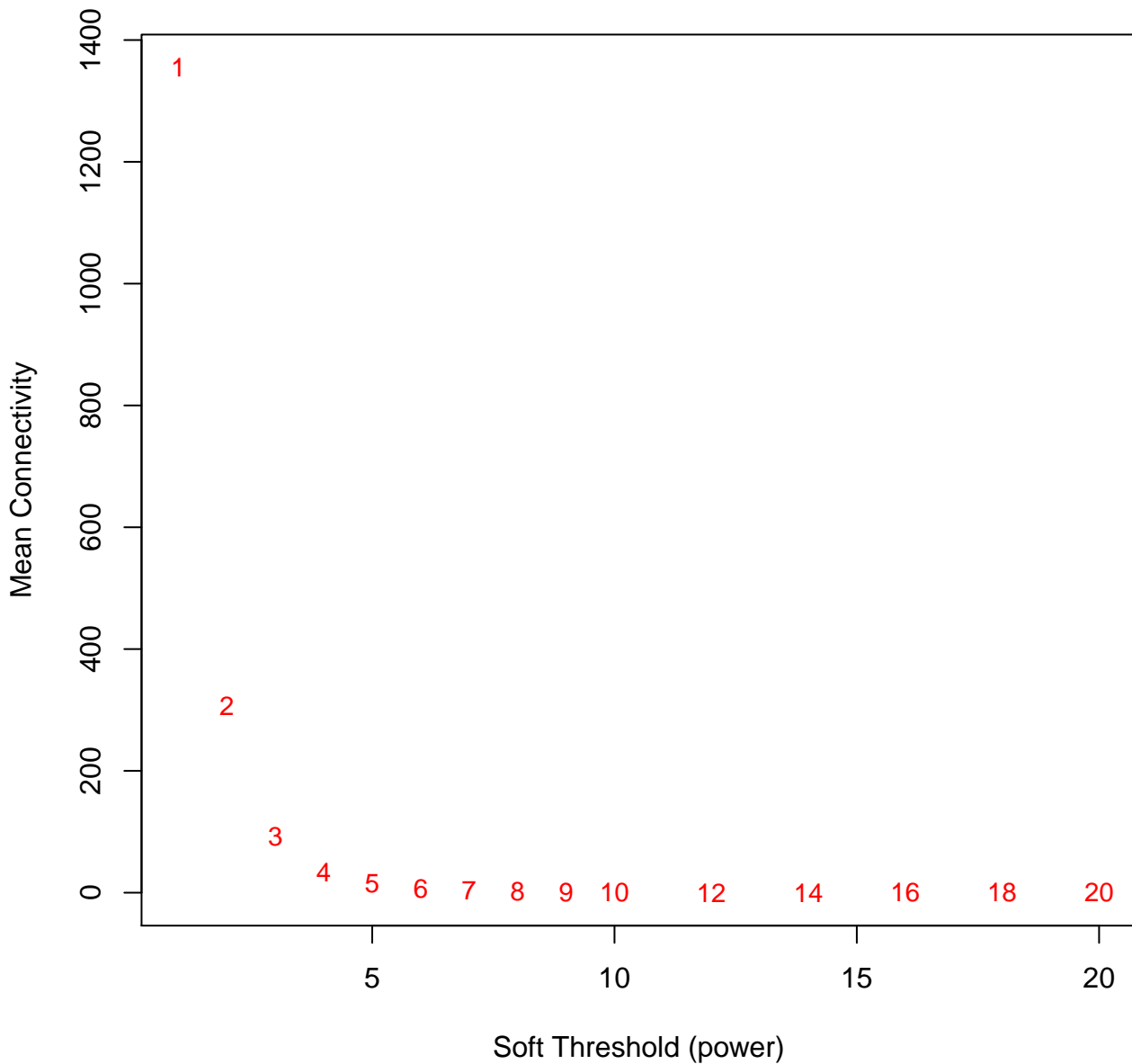

Supplement: Supplementary file 1 [file DataSheet1.zip › Supplemental Figures and Tables/Supplemental Figure 1 e and f. Scale independence and Mean connectivity for BRCA.pdf]

# Cluster dendrogram for ARCHS4

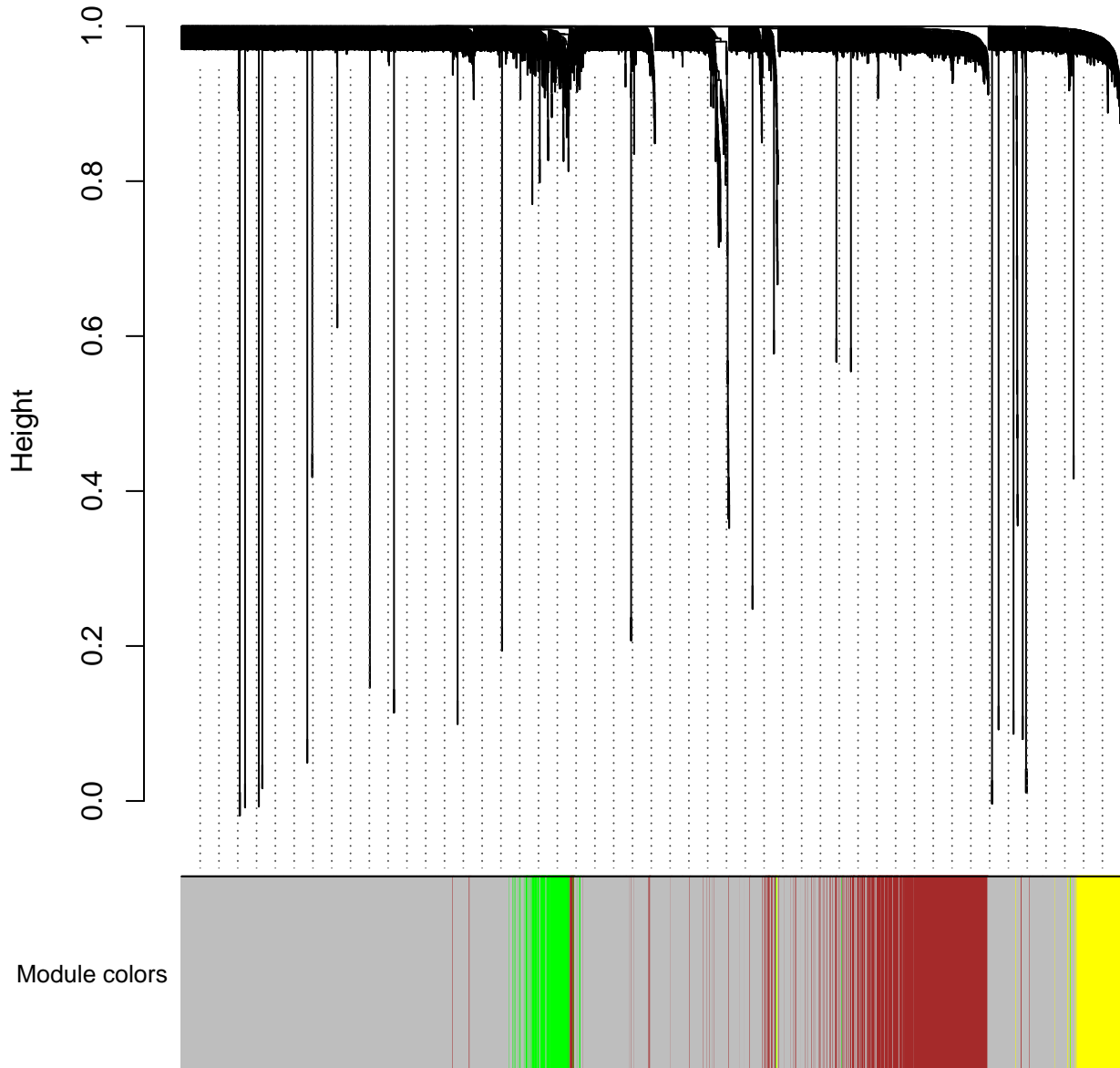

Supplement: Supplementary file 1 [file DataSheet1.zip › Supplemental Figures and Tables/Supplemental figure 3a. Cluster dendrogram for ARCHS4.pdf]

Cluster dendrogram for GSE50705

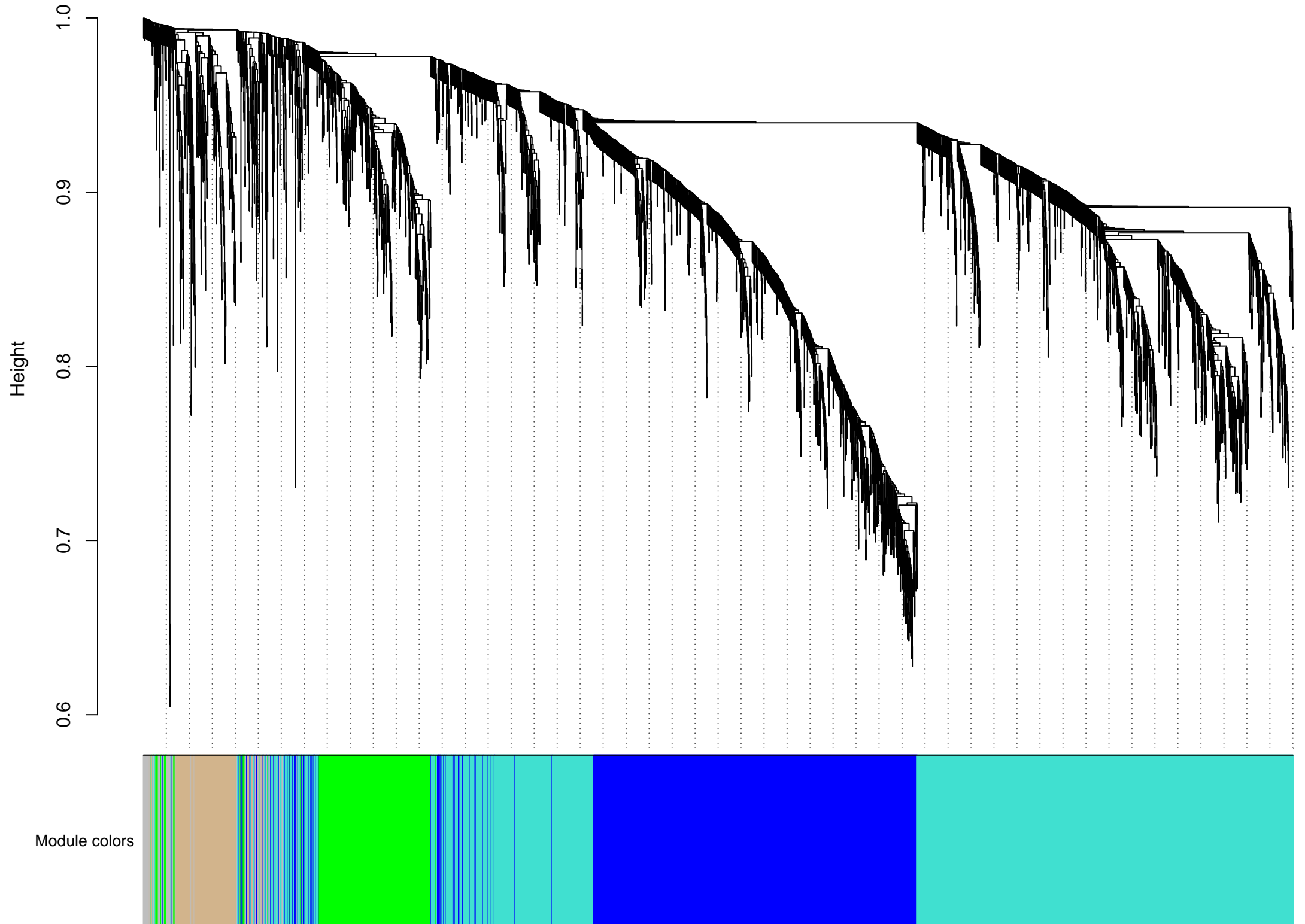

Supplement: Supplementary file 1 [file DataSheet1.zip › Supplemental Figures and Tables/Supplemental figure 3b. Cluster dendrogram for GSE50705.pdf]

Cluster dendrogram for BRCA

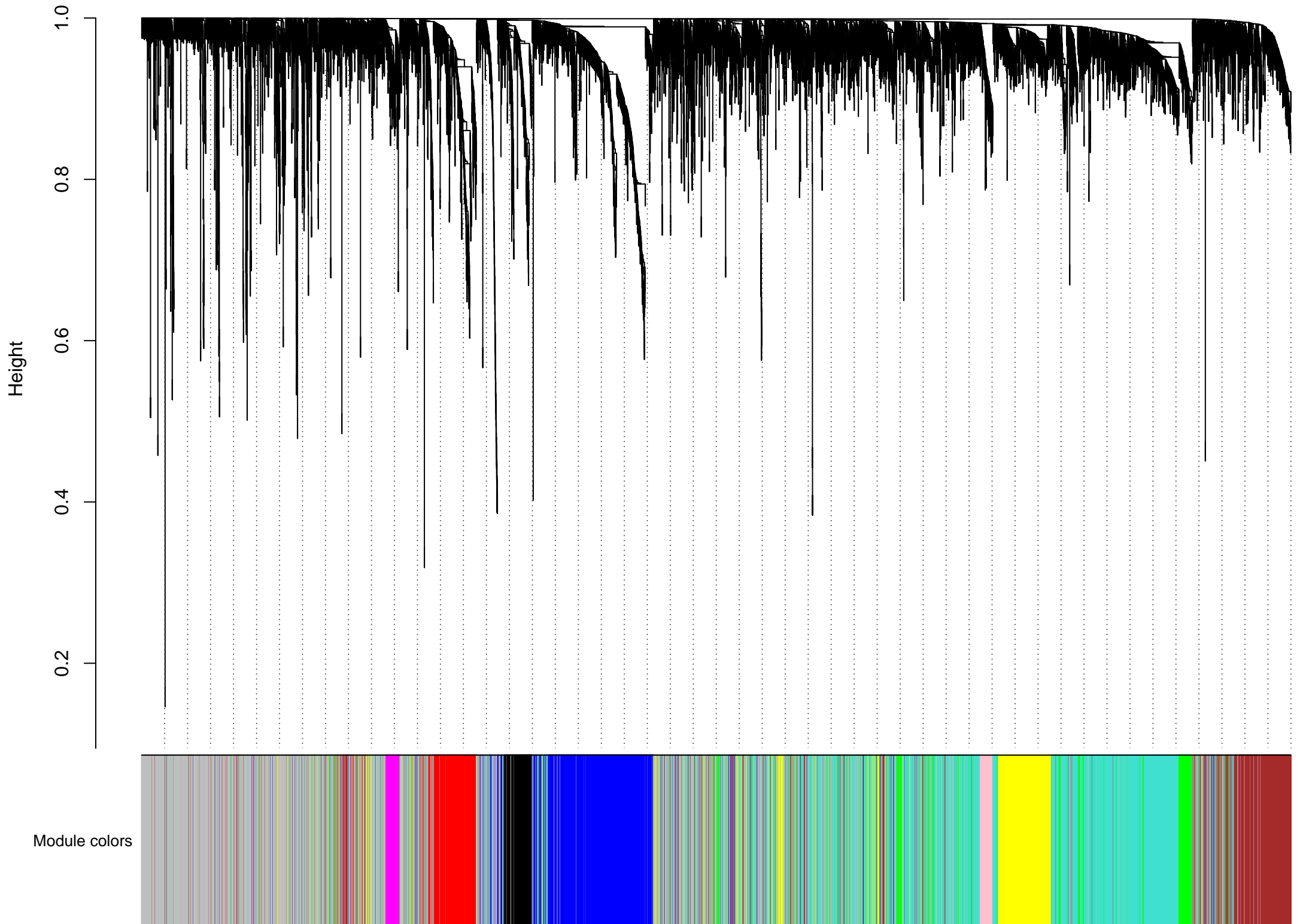

Supplement: Supplementary file 1 [file DataSheet1.zip › Supplemental Figures and Tables/Supplemental figure 3c. Cluster dendrogram for BRCA.pdf]

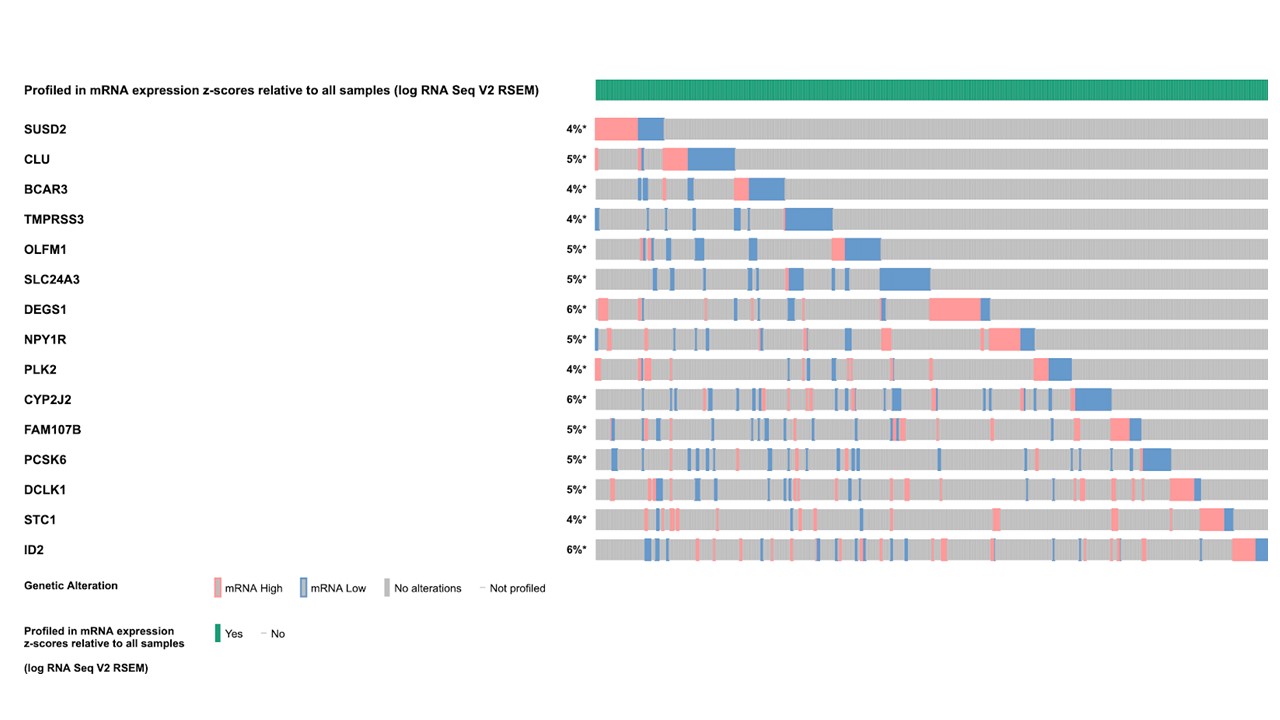

Supplement: Supplementary file 1 [file DataSheet1.zip › Supplemental Figures and Tables/Supplemental Figure 4. Transcriptional pertubations of genes with highest absoluate difference in scaled connectivity ranking.jpg]

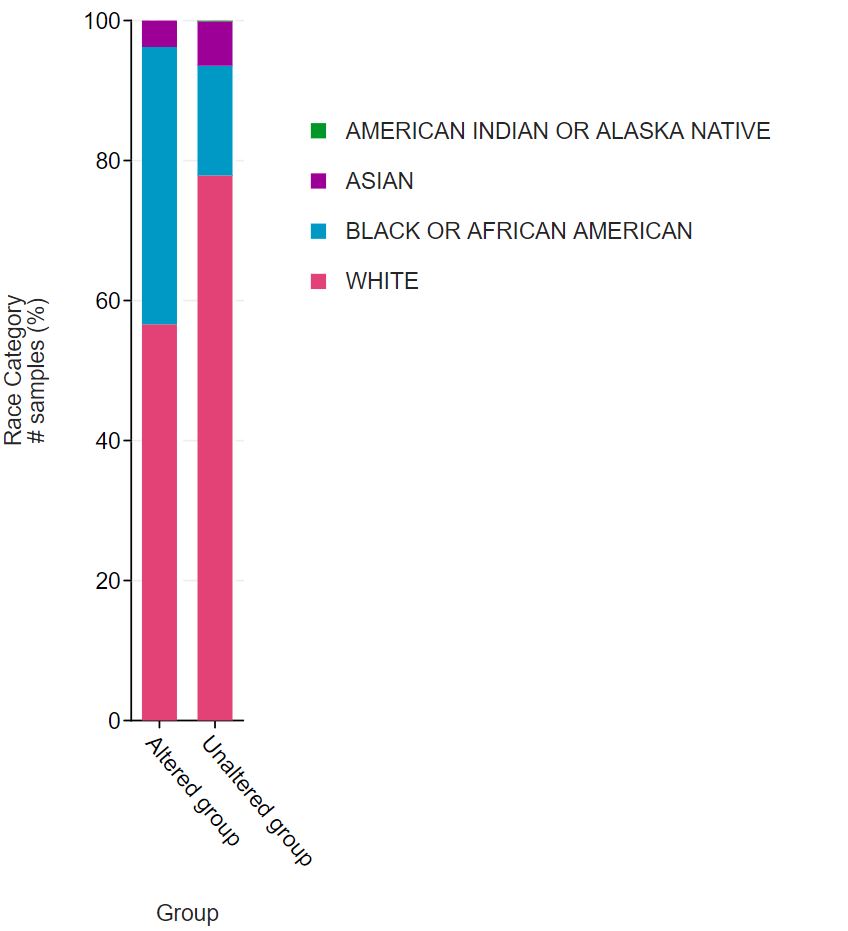

Supplement: Supplementary file 1 [file DataSheet1.zip › Supplemental Figures and Tables/Supplemental Figure 5 . Association between altered expression of CCNT1 with races.JPG]
