# Supplementary material for: Similarities and Differences in Gene Expression Networks Between the Breast Cancer Cell Line Michigan Cancer Foundation-7 and Invasive Human Breast Cancer Tissues
Source: Front Artif Intell. 2021 May 13;4:674370. doi: 10.3389/frai.2021.674370 (PMC8155268; doi:10.3389/frai.2021.674370)
Supplement: Supplementary file 1 [file Supplemental Figure 2a. Expression of genes enriched for ribosomes BRCA.pdf › Supplemental Figures and Tables/Supplemental Figure 2a. Expression of genes enriched for ribosomes BRCA.pdf]

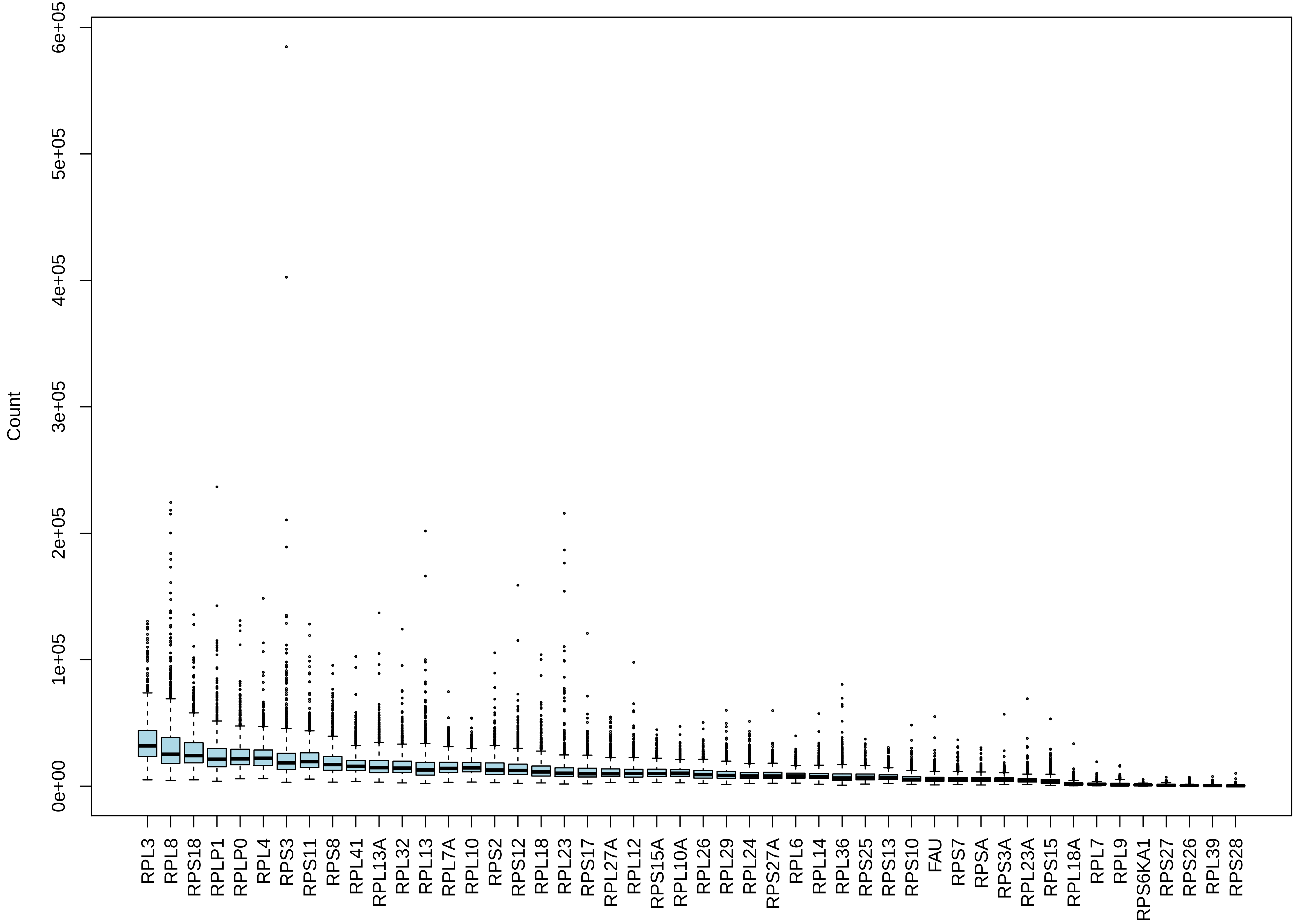

Count
0e+00
1e+05
2e+05
3e+05
4e+05
5e+05
6e+05
RPL3
RPL8
RPS18
RPLP1
RPLP0
RPL4
RPS3
RPS11
RPS8
RPL41
RPL13A
RPL32
RPL13
RPL7A
RPL10
RPS2
RPS12
RPL18
RPL23
RPS17
RPL27A
RPL12
RPS15A
RPL10A
RPL26
RPL29
RPL24
RPS27A
RPL6
RPL14
RPL36
RPS25
RPS13
RPS10
FAU
RPS7
RPSA
RPS3A
RPL23A
RPS15
RPL18A
RPL7
RPL9
RPS6KA1
RPS27
RPS26
RPL39
RPS28
